# Efficacy of Levetiracetam in Patients With Pediatric Epilepsy: A Systematic Review and Meta-Analysis

**DOI:** 10.1212/WNL.0000000000218080

**Published:** 2026-05-27

**Authors:** Simona Balestrini, Donella Puliti, Martina Lombardini, Alessandra Bettiol, Simone Gasparini, Marta Lomonaco, Elena Margherita Presotto, Elena Pia Dalmazio Tarantino, Salvatore De Masi, Renzo Guerrini

**Affiliations:** 1 Department of Neuroscience, Pharmacology and Child Health, University of Florence, Italy;; 2 Neuroscience and Human Genetics Department, Meyer Children's Hospital IRCCS, Florence, Italy;; 3 Department of Experimental and Clinical Biomedical Sciences “Mario Serio”, University of Florence, Italy;; 4 Department of Child and Adolescent Mental Health, USL Toscana Centro, Florence, Italy;; 5 Department of Neurology, Klinikum Bremen-Mitte, Gesundheit Nord gGmbH, Bremen, Germany;; 6 Research Coordination Unit, Meyer Children's Hospital IRCCS, Florence, Italy.

## Abstract

**Background and Objectives:**

Levetiracetam (LEV) is widely used in pediatric epilepsies because of its favorable pharmacokinetics, ease of administration, and perceived tolerability. However, its comparative efficacy relative to established antiseizure medications (ASMs) in children remains uncertain. We conducted a systematic review and meta-analysis of randomized controlled trials (RCTs) to evaluate LEV efficacy in pediatric epilepsies and compare outcomes vs placebo and active comparators.

**Methods:**

We systematically searched PubMed/MEDLINE and Embase (2000–6 August 2025) for RCTs enrolling patients 16 years or younger with epilepsy and reporting seizure freedom and/or ≥50% responder rate. Trials including both pediatric and adult patients were eligible if pediatric participants were represented. Comparisons included LEV vs placebo or active ASMs as monotherapy or adjunctive therapy. Primary outcomes were seizure freedom and responder rate at the trial's primary endpoint or, if not specified, longest reported follow-up. We assessed risk of bias using Cochrane Risk of Bias 2. We pooled risk differences (RDs) with 95% CIs using random-effects models, stratified by comparator and epilepsy subtype.

**Results:**

We included 25 RCTs (4,070 participants): 23 contributed to pooled meta-analyses. Across 25 trials, the mean age ranged from 0.4 to 39.3 years, reflecting pediatric-only and mixed-age RCTs; 43.8% were female. In placebo/no-therapy–controlled trials (mainly add-on studies), LEV was associated with higher seizure freedom (RD 11.0%; 95% CI 5.3%–16.7%) and responder rates (RD 24.3%; 95% CI 19.1%–29.4%). In active-comparator–controlled trials (mainly monotherapy head-to-head studies), LEV showed no overall advantage vs active comparators for seizure freedom (RD −2.4%; 95% CI −5.6% to 0.7%) or responder rate (RD −7.4%; 95% CI −23.0% to 8.1%). Fourteen trials were at high risk of bias. Sensitivity analyses confirmed benefit vs placebo but showed significant disadvantage vs active comparators in low risk-of-bias trials. Findings in pediatric-only trials (16 RCTs; 1,380 participants) were consistent with the overall results.

**Discussion:**

LEV confers benefit vs placebo, mostly as adjunctive therapy, but does not consistently outperform established ASMs in pediatric epilepsies and may be inferior in some subgroups when higher-quality evidence is considered. Limitations include substantial heterogeneity, frequent high risk of bias, variable follow-up durations, publication bias, and limited pediatric-only comparative data.

## Introduction

Levetiracetam (LEV) is a second-generation antiseizure medication (ASM) introduced in 1999 and now widely used as a broad-spectrum drug. LEV binding to presynaptic vesicle protein 2A (SV2A) has led to the suggestion that its antiseizure effect may involve modulation of presynaptic events related to vesicle release. Experimental work demonstrated that LEV may influence SV2 protein interactions and help maintain physiologic synaptic levels of SV2 and synaptotagmin, potentially reducing seizure occurrence.^1^ Despite these insights, the precise mechanism of action remains unclear.

LEV was initially approved in the United States in 1999 and in Europe in 2000 as adjunctive therapy for focal-onset seizures in patients older than 16 years and subsequently approved as monotherapy for focal epilepsy in the same population. Its indications have since expanded: it is now approved as adjunctive therapy for focal-onset seizures from 1 month of age, and for myoclonic and primary generalized tonic-clonic seizures in individuals ≥12 years with idiopathic generalized epilepsy (IGE). LEV has recently been added to the World Health Organization Essential Medicines List for children, further reinforcing its extensive adoption.^2^

Its favorable pharmacokinetic properties, availability in multiple formulations, low potential for drug-drug interactions, and relative ease of titration have likely contributed to the widespread use of LEV in clinical practice, including in pediatric populations. In addition, concerns regarding teratogenicity and long-term adverse effects associated with some first-generation ASMs may influence treatment choices, particularly in adolescents approaching reproductive age.^3,4^ These practical considerations, together with its broad regulatory indications, have supported the adoption of LEV as a commonly prescribed ASM. In a longitudinal analysis of pharmaceutical sales from 73 countries and regions, covering approximately 77% of the world's population between 2012 and 2022, LEV had the largest average yearly increase (+21.7%), becoming the second most commonly used ASM worldwide by 2022.^5^ However, phases III-IV randomized controlled trials (RCTs) have not demonstrated superiority of LEV over valproate (VPA), lamotrigine (LTG), or carbamazepine (CBZ) for generalized or focal epilepsies.^6-8^

Over the past 3 decades, second-generation ASMs have expanded therapeutic options but have not consistently improved seizure control compared with older agents.^9^ While newer ASMs may offer advantages in tolerability or pharmacokinetics, treatment decisions in pediatric epilepsy should integrate multiple factors including syndrome-specific evidence, adverse effects, drug-drug interactions, comorbidities, and patient preferences. In this context, LEV provides a useful case study to examine how evidence from randomized trials aligns with contemporary prescribing patterns. Early registration trials of LEV^10-12^ were short-term, placebo-controlled studies conducted mainly in adults with drug-resistant focal epilepsy. Although they demonstrated superiority over placebo for seizure reduction, they provided limited head-to-head comparisons with established ASMs and limited pediatric data.

To address this gap, we conducted a systematic review and meta-analysis of RCTs evaluating the efficacy of LEV in pediatric epilepsy. Our objective was to quantify seizure outcomes across epilepsy subtypes and compare LEV with placebo and active comparators, thereby providing an evidence-based assessment of its role within the broader clinical management of pediatric epilepsy.

## Methods

### Search Strategy and Selection Criteria

We included RCTs evaluating the efficacy of LEV in study populations including pediatric patients (≤16 years) with epilepsy and reporting either seizure freedom or ≥50% reduction in seizure frequency. Owing to the limited number of purely pediatric studies, we also considered mixed-age studies, from which we extracted pediatric-specific data whenever available; otherwise, we used the aggregate results of the mixed population. We excluded studies focusing exclusively on status epilepticus, neonatal, and acute symptomatic seizures.

We conducted a systematic search in the PubMed/MEDLINE and Embase electronic databases. The search included articles published from January 1, 2000, to August 6, 2025. We also screened reference lists of included studies to identify additional relevant publications. Title and abstracts were screened independently by 5 reviewers. Full-text eligibility was assessed by at least 2 of these investigators, with discrepancies resolved by consensus and adjudication by senior authors when required. The full search strategy for each database is available in the eMethods.

### Risk of Bias Assessment

Risk of bias was assessed using the Cochrane Risk of Bias 2 (RoB-2) tool,^13^ independently by 2 reviewers, with any discrepancies resolved through discussion and consensus. The criteria applied for the assessment of each domain are detailed in the eMethods.

### Data Extraction

For each included study, we extracted raw data for the number of participants who achieved the specified outcome and the total number of participants randomized to each treatment arm. Data extraction was performed by 5 researchers coauthoring this study. We resolved disagreements by consensus with senior authors. Where raw event data were not explicitly available, we calculated them from reported statistical estimates. For the 2 studies by Marson et al.,^7,8^ we derived the number of patients in 12-month remission from the Kaplan-Meier probability estimates provided in the supplementary appendix. We calculated the number of events for each arm by multiplying the probability of achieving 12-month remission at the 2-year follow-up by the total number of participants in that arm, with the result rounded to the nearest whole number.

### Outcomes

The 2 primary outcomes were seizure freedom (complete absence of seizures during the treatment period) and responder rate (≥50% reduction in seizure frequency from baseline). We analyzed seizure freedom and ≥50% responder rate as separate outcomes, as these measures are commonly used in different clinical contexts, with seizure freedom typically emphasized in newly diagnosed epilepsies and responder rate more frequently used in drug-resistant populations. If multiple time points were reported, we extracted outcomes at the trial's pre-specified primary endpoint; if not specified, we used the longest reported follow-up, to reflect the study's main efficacy assessment and avoid selective choice of earlier time points. For the primary analyses, we calculated outcome proportions using the total number of subjects randomized to each arm, consistent with an intention-to-treat (ITT) approach, irrespective of subsequent withdrawals, loss to follow-up, or protocol deviations. For trials where the original group assignment of participants lost to follow-up was not specified,^14^ we used the reported evaluable denominators (complete-case [CC] analysis). We expressed the effect size as the risk difference (RD) with 95% CIs.

### Statistical Analysis

We synthesized efficacy outcomes using random-effects models, quantified between-study heterogeneity with the *I*^2^ statistic, and visualized results using forest plots. We treated trials with a 3-arm design as separate pairwise comparisons. We visually assessed publication bias using funnel plots for all analyses, with the Egger test applied to those involving ≥10 studies. To identify potential unpublished trials, we searched ClinicalTrials.gov using the following filters: epilepsy, levetiracetam, child (birth–17 years), and completed or terminated interventional studies.

We conducted 2 prespecified subgroup analyses, stratifying results by comparator type (placebo/no-therapy–controlled vs active-comparator–controlled) and epilepsy subtype. For the latter, we categorized studies into 3 groups based on the enrolled populations: focal epilepsies (including self-limited epilepsy with centrotemporal spikes [SeLECTS], newly diagnosed, and drug-resistant focal epilepsy), IGE, and mixed or unclassified epilepsies. We used the “mixed or unclassified epilepsy” category for studies enrolling heterogeneous populations with more than 1 epilepsy type or syndrome, or where epilepsy classification was not specified, and outcomes were not reported separately. This included trials of combined focal and generalized epilepsies, unclassified epilepsies, and mixed developmental and epileptic encephalopathies. We excluded trials in infantile spasms from pooled analyses and summarized these studies descriptively because of distinct syndrome context and short follow-up. To assess the robustness of the primary findings, we performed several prespecified sensitivity analyses. First, to evaluate the potential impact of missing data on the primary ITT estimates, we conducted a CC analysis, excluding participants lost to follow-up or with missing outcome. Second, we restricted the analysis to trials enrolling exclusively pediatric patients (≤16 years) and to those judged to have low risk of bias. We performed all analyses with Stata (version 16.1, StataCorp., College Station, TX).

### Standard Protocol Approvals, Registrations, and Patient Consents

This systematic review and meta-analysis were conducted in accordance with the Preferred Reporting Items for Systematic reviews and Meta-Analyses (PRISMA) guidelines. The study protocol was registered on PROSPERO (CRD420251104042). As this research involved only published data from previously conducted trials, formal approval from an institutional ethics committee and informed consent from participants were not required.

### Data Availability

All data included in this meta-analysis are publicly available from the original published studies. All relevant data are reported in Article, eMethods, or eTable 1.

## Results

**Figure 1:**
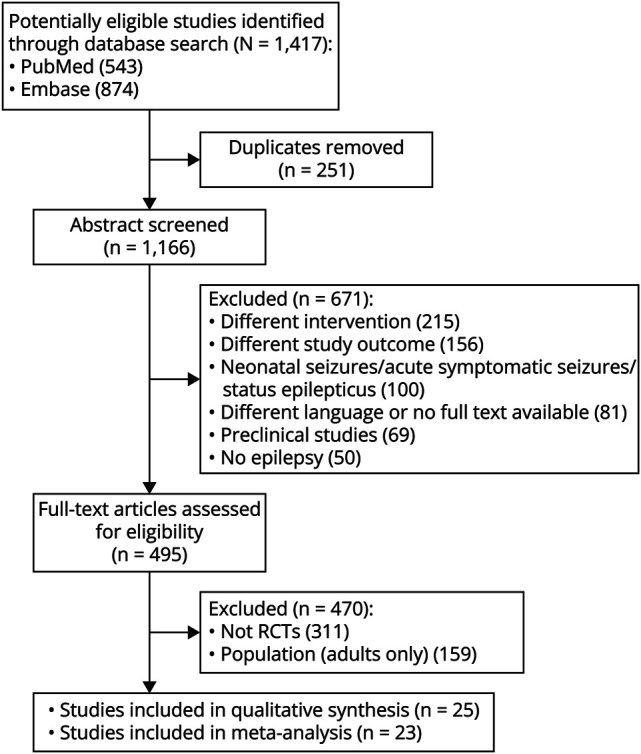
PRISMA Flow Diagram for Studies Included in the Meta-Analysis PRISMA = Preferred Reporting Items for Systematic reviews and Meta-Analyses; RCT = randomized controlled trial.

The initial literature search identified 1,417 potentially relevant records from PubMed (n = 543) and Embase (n = 874). After the removal of 251 duplicate records, we screened 1,166 abstracts for eligibility. We then excluded 671 records, as they investigated a different intervention (n = 215), reported irrelevant outcomes (n = 156), or those focused exclusively on status epilepticus or acute symptomatic seizures (n = 100). This process left 495 full-text reports that were assessed for eligibility. Of these, we excluded a further 470 because they were not RCTs (n = 311) or exclusively enrolled adult patients (n = 159) (see eTable 1 for details). Ultimately, we included 25 studies in the qualitative synthesis. Of these, 23 contributed data to the quantitative meta-analysis; 2 infantile spasms trials were summarized descriptively. The study selection process is detailed in the PRISMA flow diagram (Figure 1).

The 25 studies enrolled 4,070 patients and were published between 2006 and 2025.^7,8,14-36^ The main characteristics of the trials are summarized in Table 1. The trials originated from different geographic regions, with a marked increase in publications, particularly from India and Iran, since 2020. To distinguish between the 2 distinct trials reported by Marson et al. in 2021^7,8^ within the SANAD II program, we adopted the labels “Marson et al., 2021a” for the trial on focal epilepsy^7^ and “Marson et al., 2021b” for that on generalized and unclassified epilepsy.^8^

**Table 1:** Characteristics of 25 Included Studies

Study (author, year)	Country	Epilepsy type	Patients, n	Age range	Follow-up	Type of treatment	Comparator	Meta-analysis grouping	Blinding	Funding	Seizure freedom	Responder rate (≥50%)
Glauser (2006)^15^	USA	Focal-drug resistant	198	4–16 y	3.5 mo	ADJ	Placebo	Placebo/no-therapy	Double-blind	Industry	^a^	^a^
Berkovic (2007)^16^	Australia	Generalized-IGE	164	4–65 y	6 mo	ADJ	Placebo	Placebo/no-therapy	Double-blind	Industry	^a^	^a^
Coppola (2007)^17^	Italy	Focal-SeLECTs	39	3–12 y	18.5 mo	MNT	OXC	Active comparator	Open-label	Not reported	^a^	
Noachtar (2008)^18^	Germany	Generalized-IGE	122	12–65 y	4 mo	ADJ	Placebo	Placebo/no-therapy	Double-blind	Industry	^a^	^a^
Levisohn (2009)^19^	USA	Focal-drug resistant	98	4–16 y	3 mo	ADJ	Placebo	Placebo/no-therapy	Double-blind	Industry	^a^	^a^
Peltola (2009)^20^	Finland	Focal-drug resistant	158	12–70 y	3 mo	ADJ	Placebo	Placebo/no-therapy	Double-blind	Industry	^a^	^a^
Piña-Garza (2009)^21^	USA	Focal-drug resistant	116	1–48 mo	0.2 mo	ADJ	Placebo	Placebo/no-therapy	Double-blind	Industry	^a^	^a^
Fattore (2011)^22^	Italy	Generalized-IGE	59	4–16 y	0.5 mo	MNT	Placebo	Placebo/no-therapy	Double-blind	Industry	^a^	^a^
Rosenow (2012)^23^	Germany	Mixed	409	12–84 y	6.5 mo	MNT	LTG	Active comparator	Open-label	Industry	^a^	
Borggraefe (2013)^24^	Germany	Focal-SeLECTs	44	6–12 y	6 mo	MNT	STM	Active comparator	Double-blind	Industry	^a^	
Mahmoud (2013)^25,b^	Saudi Arabia	Generalized-spasms	20	0–24 mo	1 mo	MNT	TPM	^b^	Open-label	Independent		^a^
Jung (2015)^26^	Korea	Focal-newly diagnosed	121	4–16 y	12 mo	MNT	CBZ	Active comparator	Open-label	Independent	^a^	^a^
Akhondian (2020)^27^	Iran	Focal-newly diagnosed	50	1–16 y	6 mo	MNT	CBZ	Active comparator	Single-blind	Industry	^a^	
Ahadi (2020)^28^	Iran	Focal-SeLECTs	92	4–12 y	6 mo	MNT	CBZ	Active comparator	Open-label	Independent	^a^	
Liu (2020)^29^	China	Mixed	100	11–40 mo	3 mo	ADJ	None	Placebo/no-therapy	Not reported	Independent	^a^	^a^
Manreza (2021)^30^	Brazil	Focal-drug resistant	126	4–65 y	4 mo	ADJ	Placebo	Placebo/no-therapy	Double-blind	Industry	^a^	^a^
Marson (2021a)^7^	UK	Focal-newly diagnosed	990	5–91 y	24 mo	MNT	ZNS or LTG	Active comparator	Open-label	Independent	^a^	
Marson (2021b)^8^	UK	Mixed	520	5–94 y	24 mo	MNT	VPA	Active comparator	Open-label	Independent	^a^	
Santhosh (2021)^31^	India	Focal-newly diagnosed	99	12–55 y	6 mo	MNT	CBZ	Active comparator	Open-label	Independent	^a^	
Suo (2021)^32^	China	Focal-SeLECTs	70	6–10 y	6 mo	MNT	OXC	Active comparator	Not reported	Independent	^a^	
Archna (2022)^33^	India	Mixed	101	2–12 y	3 mo	ADJ	MAD	Active comparator	Open-label	Independent	^a^	^a^
Daneshyar (2022)^14^	Iran	Generalized-IGE	102	14–41 y	12 mo	MNT	VPA or LTG	Active comparator	Double-blind	Independent		^a^
Gowda (2022)^34,b^	India	Generalized-spasms	100	2–60 mo	0.5 mo	ADJ	None	^b^	Not reported	Independent	^a^	^a^
Montazerlotfelahi (2024)^35^	Iran	Focal-newly diagnosed	120	2–14 y	6 mo	MNT	CBZ	Active comparator	Double-blind	Independent	^a^	
Meena (2025)^36^	India	Mixed	52	1–18 y	6 mo	MNT	VPA	Active comparator	Open-label	Not reported	^a^	

Abbreviations: ADJ = adjunctive; CBZ = carbamazepine; IGE = idiopathic generalized epilepsy; ITT = intention-to-treat; LTG = lamotrigine; MAD = modified Atkins diet; MNT = monotherapy; OXC = oxcarbazepine; PP = per-protocol; SeLECTS = self-limited epilepsy with centrotemporal spikes; STM = sulthiame; TPM = topiramate; VPA = valproate; ZNS = zonisamide.

The term “None” in the Comparator column refers to studies in which the control group received standard therapy alone, without any adjunctive treatment or placebo administration.

a Data for this outcome were available in the study (either explicitly reported or derivable).

b These studies were included in the qualitative synthesis but excluded from the meta-analysis because infantile spasms represent a distinct clinical entity with specific treatment guidelines and shorter evaluation periods, precluding a direct comparison with other epilepsy subtypes.

**Figure 2:**
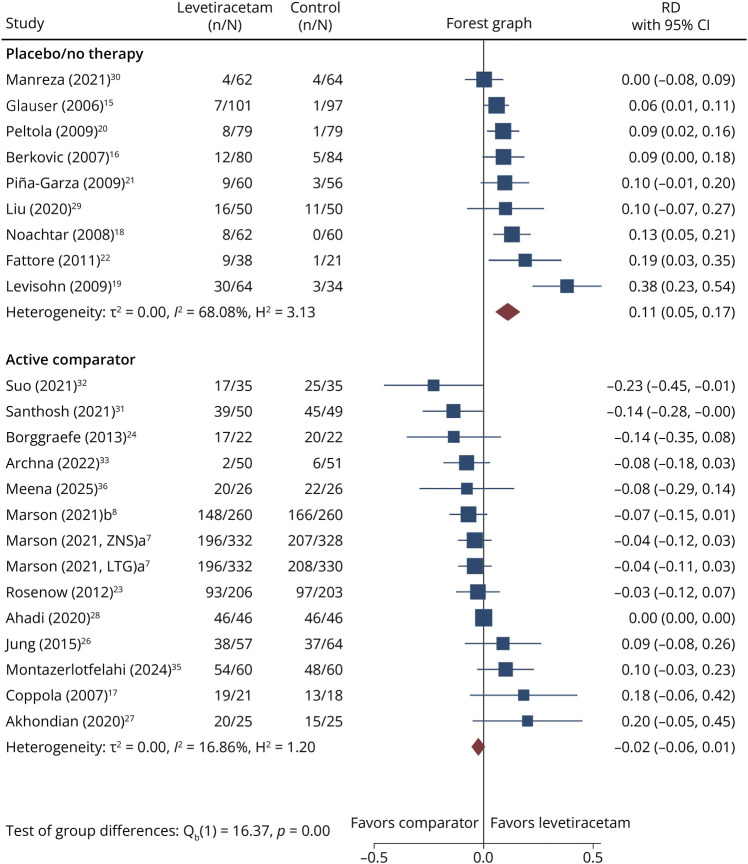
Forest Plot Showing the RD in the Proportion of Seizure-Free Patients Between Levetiracetam and the Comparator Arm Positive values indicate a higher proportion of seizure-free patients with levetiracetam, whereas negative values indicate a higher proportion with the comparator. Studies are ordered by effect size (from smallest to largest). The size of each square is proportional to the study weight in the random-effects model, and the horizontal lines represent 95% CIs. The diamond represents the overall pooled estimate. RD = risk difference.

**Figure 3:**
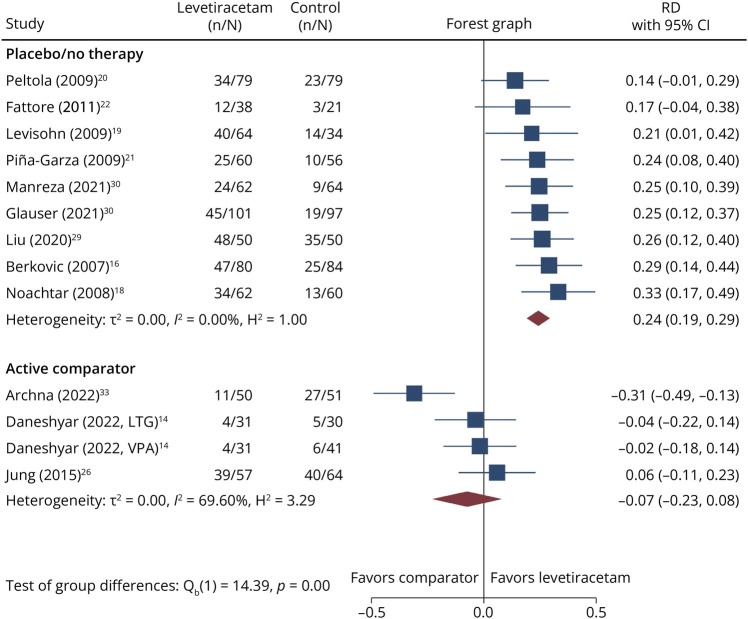
Forest Plot Showing the RD in the Proportion of Responder Between Levetiracetam and the Comparator Arm Positive values indicate a higher proportion of responder with levetiracetam, whereas negative values indicate a higher proportion with the comparator. Studies are ordered by effect size (from smallest to largest). The size of each square is proportional to the study weight in the random-effects model, and the horizontal lines represent 95% CIs. The diamond represents the overall pooled estimate. RD = risk difference.

Marked heterogeneity emerged across trials, both in study populations and design. Regarding epilepsy type, 14 trials enrolled patients with focal epilepsy, 6 with generalized epilepsy, and 5 with mixed epilepsies. Patients age also varied considerably: 16 studies focused exclusively on children and 9 included both pediatric and adult populations. Since none of the 9 mixed-age trials identified provided age-stratified efficacy outcomes for the pediatric subgroup, we used aggregate data for the total study populations in the meta-analysis. For comparators, most trials (n = 14) evaluated LEV monotherapy against other commonly used ASMs, such as CBZ and VPA. A single trial^22^ investigated LEV monotherapy vs placebo. Ten trials assessed LEV as adjunctive therapy; of these, 7 trials compared it with placebo, 2 trials^29,34^ used standard therapy alone without placebo, and 1 trial^33^ used the modified Atkins diet. For the subgroup analyses presented in Figures 2–3 and Table 2, we categorized trials as placebo/no-therapy–controlled if the control arm received placebo or standard therapy alone without an active head-to-head comparator, and as active-comparator–controlled if LEV was directly compared with another ASM or active intervention. We applied this classification irrespective of whether LEV was administered as monotherapy or adjunctive therapy.

**Table 2:** Pooled Efficacy and Sensitivity Analyses of Levetiracetam for Seizure Freedom and Responder Rate, Stratified by Epilepsy Type and Comparator (23 Studies)

Epilepsy subgroup/analysis type	Seizure freedom		Responder rate (≥50%)	
	vs Placebo/no-therapy	vs Active comparator	vs Placebo/no-therapy	vs Active comparator
	Pooled RD (95% CI) (no. of studies [comparisons], patients, *I*^2^)	Pooled RD (95% CI) (no. of studies [comparisons], patients, *I*^2^)	Pooled RD (95% CI) (no. of studies [comparisons], patients, *I*^2^)	Pooled RD (95% CI) (no. of studies [comparisons], patients, *I*^2^)
Main analysis (ITT)				
Focal-SeLECTS	—	−4.1% (−18.5% to 10.2%) 4 studies, 245 patients, *I*^2^ = 40.5%	—	—
Focal-newly diagnosed	—	0.2% (−7.6% to 8.0%) 5 (6) studies, 1,712 patients, *I*^2^ = 60.9%	—	5.9% (−11.0% to 22.8%) 1 study, 121 patients
Focal-drug resistant	11.3% (0.1% to 22.5%) 5 studies, 696 patients, *I*^2^ = 88.5%	—	22.0% (15.2% to 28.8%) 5 studies, 696 patients, *I*^2^ = 0.0%	—
Generalized-IGE	12.2% (6.4% to 18.0%) 3 studies, 345 patients, *I*^2^ = 0.0%	—	28.0% (18.4% to 37.6%) 3 studies, 345 patients, *I*^2^ = 0.0%	−2.6% (−14.5% to 9.3%) 1 (2) study, 133 patients
Mixed	10.0% (−7.3% to 27.3%) 1 study, 100 patients	−5.9% (−11.2% to −0.7%) 4 studies, 1,082 patients, *I*^2^ = 0.0%	26.0% (12.2% to 39.8%) 1 study, 100 patients	−30.9% (−48.8% to −13.1%) 1 study, 101 patients
Total (ITT)	11.0% (5.3% to 16.7%) 9 studies, 1,141 patients, *I*^2^ = 68.1%	−2.4% (−5.6% to 0.7%) 13 (14) studies, 3,039 patients, *I*^2^ = 16.9%	24.3% (19.1% to 29.4%) 9 studies, 1,141 patients, *I*^2^ = 0.0%	−7.4% (−23.0% to 8.1%) 3 (4) studies, 355 patients, *I*^2^ = 69.6%
Sensitivity analyses				
Overall (complete case)	12.9% (5.1% to 20.8%) 9 studies, 1,029 patients, *I*^2^ = 80.4%	−2.7% (−5.8% to 0.4%) 13 (14) studies, 2,733 patients, *I*^2^ = 24.0%	25.8% (20.4% to 31.2%) 9 studies, 1,029 patients, *I*^2^ = 0.0%	−9.8% (−22.8% to 3.1%) 3 (4) studies, 304 patients, *I*^2^ = 70.2%
Pediatric trials	15.5% (4.3% to 26.6%) 5 studies, 571 patients, *I*^2^ = 77.3%	0.1% (−7.0% to 7.3%) 9 studies, 689 patients, *I*^2^ = 37.7%	23.7% (16.7% to 30.8%) 5 studies, 571 patients, *I*^2^ = 0.0%	−12.4% (−48.5% to 23.7%) 2 studies, 222 patients, *I*^2^ = 88.4%
Low-risk-of-bias trials	10.2% (5.5% to 14.9%) 3 studies, 444 patients, *I*^2^ = 0.0%	−4.8% (−9.3% to −0.4%) 2 (3) studies, 1,842 patients, *I*^2^ = 0.0%	25.1% (13.7% to 36.6%) 3 studies, 444 patients, *I*^2^ = 41.4%	—

Abbreviations: *I*^2^ = I-squared statistic for heterogeneity; IGE = idiopathic generalized epilepsy; ITT = intention-to-treat; RD = risk difference; SeLECTS = self-limited epilepsy with centrotemporal spikes.

The number of studies is reported, with the number of comparisons shown in parentheses when different (due to multiarm trials). A dash (—) indicates that no studies were available for that specific subgroup and comparator. The patient count reflects the sum of participants across all comparisons included in the meta-analysis, not the number of unique patients. Consequently, participants in the common control arms of multi-arm trials were counted multiple times. Placebo/no-therapy includes placebo-controlled trials and trials in which the control arm received standard therapy alone without an active comparator.

Outcome definitions and follow-up duration also varied widely. Nearly all studies (n = 23) reported the proportion of seizure-free patients whereas only 14 reported the responder proportion (≥50% reduction). Follow-up duration varied widely across studies, ranging from 5 days to 18.5 months. Three trials^21,22,34^ had very short evaluation periods of 2 weeks or less while several trials, particularly those in focal epilepsy, extended follow-up to 12–18 months.

Funding sources varied across the 25 trials, with 11 supported by the pharmaceutical industry and 12 by independent sources. For 2 studies,^17,36^ the funding source was not reported, and our attempts to contact the corresponding authors for clarification were unsuccessful. There was a strong association between the source of funding and the choice of comparator (*p* = 0.006). Most industry-funded trials compared LEV against a placebo (73%; 8 of 11), while independently funded trials mostly used an active comparator (83%; 10 of 12). A temporal trend was also apparent: trials published before 2020 were largely industry-funded, whereas most studies published since 2020 were independently funded.

### Quality Evaluation

**Figure 4:**
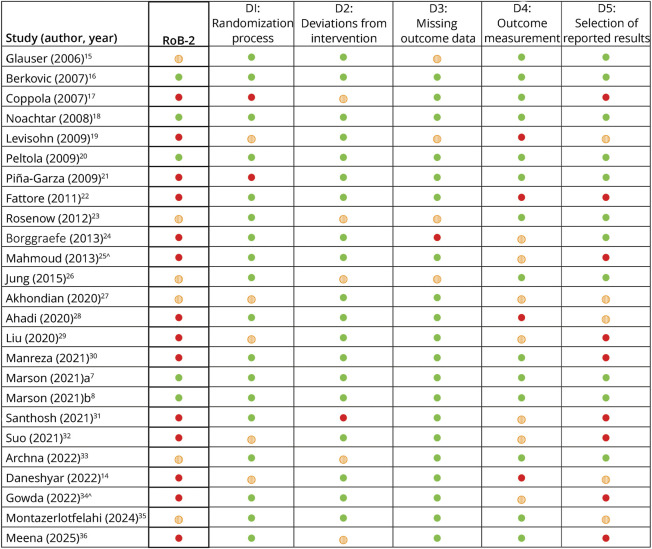
Risk of Bias Assessment of 25 Included Studies According to RoB 2 Domains ● = High risk; ◍ = Some concerns; ● = Low risk. RoB-2 = Cochrane Risk of Bias 2 tool. ^These studies were included in the qualitative synthesis but excluded from the meta-analysis because infantile spasms represent a distinct clinical entity with specific treatment guidelines and shorter evaluation periods, precluding a direct comparison with other epilepsy subtypes.

Most studies were considered at high risk of bias according to the RoB-2 tool, with 14 studies classified as high risk, 6 raising some concerns, and only 5 judged to be at low risk (Figure 4). High-risk judgments were primarily due to limitations in the method of data collection of seizure frequency (domain 4) and in the selection of the reported results (domain 5). Specifically, we rated to be at high risk for domain 4 those studies in which seizure frequency was reconstructed retrospectively during follow-up visits without the use of daily seizure diaries completed by parents or caregivers^14,19,28^ or collected using a semiquantitative scale.^22^ Regarding domain 5, 7 studies^17,25,29-32,34^ did not report protocol registration, 1 study^36^ had an invalid registration number, and 1 study^22^ had a registered protocol that completely lacked information on study outcomes.

### Pooled Analyses

Figures 2 and 3 show the forest plots of seizure-free and responder proportions stratified by type of comparator (placebo/no-therapy vs active comparator), irrespective of whether LEV was used as monotherapy or adjunctive therapy. Among the 23 trials contributing to the meta-analysis, the number of studies included in each forest plot varied according to outcome reporting. In placebo/no-therapy–controlled trials (9 trials, of which 8 evaluated adjunctive therapy), LEV demonstrated a statistically significant benefit, with an overall RD of 11.0% (95% CI 5.3%–16.7%) for seizure freedom and 24.3% (95% CI 19.1%–29.4%) for responder rate. In active-comparator–controlled trials (predominantly monotherapy head-to-head studies), LEV showed no overall benefit for both seizure freedom (13 trials, of which 12 evaluated monotherapy, RD = −2.4%; 95% CI −5.6% to 0.7%) and ≥50% seizure frequency reduction (3 trials, of which 2 evaluated monotherapy; RD = −7.4%; 95% CI −23.0% to 8.1%). The difference between comparator subgroups was statistically significant for both seizure freedom (*p* < 0.001) and responder rate (*p* < 0.001).

Because placebo/no-therapy–controlled trials were predominantly adjunctive therapy studies in drug-resistant populations, these pooled estimates primarily reflect the add-on efficacy of LEV. Conversely, because active-comparator trials were predominantly monotherapy head-to-head studies, these pooled estimates primarily reflect the comparative effectiveness of LEV as monotherapy. In this context, LEV did not demonstrate superiority over established ASMs for seizure freedom. Responder rate outcomes were infrequently reported in monotherapy head-to-head trials, limiting the precision of pooled estimates for this endpoint in the active-comparator subgroup.

Substantial heterogeneity was observed in specific subgroups (*I*^2^ = 68.1% for seizure freedom in placebo/no-therapy–controlled trials, and 69.6% for responder rate in active-comparator–controlled trials), suggesting that the effect of LEV may vary across studies and that a single pooled estimate should be interpreted with caution. To investigate the sources of this heterogeneity, we further stratified the 2 main comparison groups, placebo/no-therapy vs active comparator, by epilepsy type.

Table 2 summarizes the trials included in the meta-analysis, reporting the proportions of seizure-free and responder patients for each treatment arm, stratified by epilepsy type and type of comparator.

For each study group, pooled RD (95% CI), number of studies, number of enrolled patients, and *I*^2^ statistic are reported. In placebo/no-therapy–controlled trials, LEV demonstrated a significant benefit in drug-resistant focal epilepsy and in IGE for both seizure-free and responder rate outcomes. Specifically, in drug-resistant focal epilepsy, the pooled RD was 11.3% (95% CI 0.1%–22.5%) for seizure freedom and 22.0% (95% CI 15.2%–28.8%) for responder rate. In IGE, LEV showed an RD of 12.2% (95% CI 6.4%–18.0%) for seizure freedom and 28.0% (95% CI 18.4%–37.6%) for responder rate. In active-comparator–controlled trials, LEV showed no meaningful effect in focal-SeLECTS (RD = −4.1% in seizure freedom) or in newly diagnosed focal epilepsy (RD 0.2% and 5.9%), whereas in the mixed epilepsy subgroup, the effect was significantly negative for both outcomes (RD = −5.9% for seizure freedom and RD = −30.9% for responder rate), indicating a disadvantage of LEV compared with the active comparator.

### Sensitivity Analysis

Sensitivity analyses, summarized in the lower section of Table 2, confirmed that LEV consistently showed a clear advantage vs placebo/no-therapy but a significant negative effect vs active comparators in trials at low risk of bias. The CC analysis, which excluded participants lost to follow-up, yielded results highly consistent with the primary ITT estimates across all outcomes. For instance, the pooled RD for seizure freedom vs placebo/no-therapy was 12.9% (95% CI 5.1%–20.8%) in the CC analysis, compared with 11.0% (95% CI 5.3%–16.7%) in the primary analysis, with largely overlapping confidence intervals. Similarly, the responder rate and comparisons against active comparators remained stable in the CC-based model. The analysis restricted to the 16 pediatric trials (1,380 patients) showed a consistent benefit for LEV over placebo (RD for seizure freedom 15.5%; RD for responder rate 23.7%) but not over active comparators (RD for seizure freedom 0.1%; RD for responder rate −12.4%). In the analysis restricted to the 5 low risk-of-bias trials (1,954 patients), a benefit was again confirmed against placebo/no-therapy (RD for seizure freedom 10.2%; RD for responder rate 25.1%), but LEV showed a statistically significant negative effect vs active comparators (RD for seizure freedom −4.8%; 95% CI −9.3% to −0.4%). No active-comparator–controlled trials reporting responder rate could be rated as low risk of bias. Overall, the consistency across these sensitivity analyses reinforces the robustness of our findings, confirming that the study conclusions remain stable regardless of data-related assumptions or variations in study characteristics.

### Publication Bias

**Figure 5:**
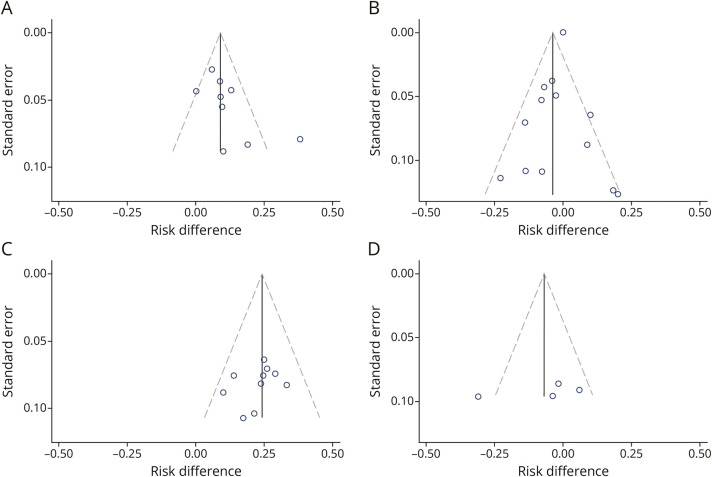
Funnel Plots for Publication Bias Assessment (A) Seizure freedom in placebo/no-therapy-controlled trials (Egger test: *p* = 0.017). (B) Seizure freedom in active-comparator–controlled trials (Egger test: *p* = 0.99). (C) Responder rate in placebo/no-therapy–controlled trials (Egger test: *p* = 0.61). (D) Responder rate in active-comparator–controlled trials (Egger test was not performed as the analysis included fewer than 10 studies).

Funnel plots for each outcome, stratified by comparator type, are shown in Figure 5. Significant right-sided asymmetry was observed for seizure freedom in placebo/no-therapy–controlled trials (*p* = 0.017), while no evidence of bias was found in active-comparator–controlled trials (*p* = 0.99). Regarding responder rate, no significant asymmetry was detected in placebo/no-therapy–controlled studies (*p* = 0.61). Finally, the Egger test was not performed for the responder rate in active-comparator–controlled trials due to the limited number of studies (n < 10). Furthermore, our systematic search for unpublished protocols on ClinicalTrials.gov identified 1 eligible placebo-controlled RCT (NCT01392768, “Efficacy and Safety of Levetiracetam in Partial Seizures Control, With or Without Secondary Generalization”; n = 126; age range 4–65 years) registered in 2011 that has not been published to date.

### Infantile Spasms

Finally, regarding infantile spasms, the 2 excluded trials^25,34^ were analyzed qualitatively. In the first study^25^ (n = 20), LEV was compared with topiramate as a second-line treatment, showing no significant difference in responder rates (RD = 2.0%). In the second trial^34^ (n = 100), the addition of LEV to standard adrenocorticotropic hormone (ACTH), therapy was compared with ACTH alone. This study reported no difference in seizure freedom (RD = 0.0%) and a slightly lower overall responder rate in the LEV group (RD = −6.0%). Both studies were judged to be at high risk of bias across almost all assessed domains.

## Discussion

In this systematic review and meta-analysis of 25 RCTs including 4,070 patients, we found that LEV offers benefit vs placebo/no-therapy, which largely evaluated adjunctive use in drug-resistant epilepsy. However, in active-comparator–controlled trials, predominantly monotherapy head-to-head studies, LEV did not show superiority over established ASMs and, in analyses restricted to trials at low-risk-of-bias appeared inferior for seizure freedom. In placebo/no-therapy–controlled trials, mainly add-on, LEV was associated with a statistically significant increase in both seizure freedom and responder rate. Overall, the pooled RD was 11.0% for seizure freedom and 24.3% for responder rate, indicating a benefit over placebo/no-therapy. These effects were particularly evident in drug-resistant focal epilepsy and IGE, where RDs ranged from about 11%–12% for seizure freedom and 22%–28% for responder rate. By contrast, when LEV was compared with active comparators, no overall benefit emerged. The pooled RD was −2.4% for seizure freedom and −7.4% for responder rate, indicating that LEV is, at best, similar in efficacy to established ASMs and may be less effective in certain contexts. For instance, in the mixed epilepsy subgroup, LEV showed a statistically significant disadvantage compared with active comparators for both seizure freedom (RD −5.9%) and responder rate (RD −30.9%). These findings should be interpreted with caution as this subgroup included heterogeneous populations and unclassified epilepsies, and therefore, they do not reflect a single, well-defined clinical syndrome. In focal epilepsies, the SANAD II trial showed that LEV was neither clinically nor cost-effective compared with both LTG and ZNS as initial monotherapy for newly diagnosed focal epilepsy.^7^ In generalized and unclassified epilepsies, SANAD II demonstrated VPA to be superior to LEV.^8^ Although these 2 large, pragmatic, independently funded trials largely drive the negative or neutral estimates in active-comparator subgroups, we show that our results remain stable even when the analysis does not include them.

In SeLECTS, active comparators were frequently oxcarbazepine or CBZ. Although sodium channel blockers have been associated in rare cases with increased sleep-activated epileptiform activity or epileptic encephalopathy with spike-wave activation in sleep,^37^ this appears uncommon.^38^ Moreover, SeLECTS is typically self-limited and treatment may not be required in all patients.^39^

Our risk-of-bias assessment revealed major methodological limitations in the LEV literature. Only 5 of the 25 RCTs were judged to be at low risk of bias, whereas 14 were at high risk, often due to suboptimal outcome measurement and selective reporting. Seizure frequency was sometimes reconstructed retrospectively or recorded with semiquantitative scales rather than systematically documented via daily seizure diaries, leading to a high risk of measurement bias (domain 4). In addition, many studies lacked prospective trial registration, had invalid registration numbers, or provided insufficiently detailed protocols (domain 5), limiting our ability to rule out selective reporting of favorable outcomes.

Prespecified sensitivity analyses supported the robustness of our findings. The CC analysis, which excluded participants lost to follow-up, was highly consistent with the primary ITT estimates. Restricting the analysis to pediatric-only trials confirmed a clear benefit vs placebo/no-therapy but no advantage vs active comparators. When the analysis was limited to trials at low risk of bias (n = 5, 1,954 patients), LEV again showed benefit compared with placebo/no-therapy, but a statistically significant negative effect vs active comparators for seizure freedom (RD −4.8%). The right-sided asymmetry for seizure freedom in placebo/no-therapy–controlled trials, together with the identified unpublished eligible RCT (NCT01392768), raises the possibility of publication bias in this subset of studies. This trial was also identified in the Cochrane review^40^ on add-on LEV in drug-resistant focal epilepsy as a potentially eligible but unpublished study. However, funnel plot asymmetry and Egger test results should be interpreted cautiously, as they may reflect heterogeneity, small study effects, or other sources of variability. No evidence of asymmetry was observed in active-comparator trials, although the limited number of studies for some outcomes reduces the power to detect bias.

Heterogeneity was substantial in several pooled analyses, particularly in placebo/no-therapy–controlled trials of focal epilepsy, reflecting differences in epilepsy type and etiology, age range, follow-up duration, dosing regimens, and outcome definitions. This heterogeneity underscores that pooled estimates should be interpreted cautiously and in a syndrome-specific manner. To support clinically appropriate interpretation, we analyzed seizure freedom and ≥50% responder rate as distinct outcomes and performed prespecified subgroup analyses by epilepsy type (focal, IGE, and mixed/unclassified). However, not all trials reported both outcomes, and the mixed/unclassified category included heterogeneous populations; these factors limited the precision and comparability of some subgroup estimates.

Overall, LEV did not demonstrate superior efficacy to active comparators across pediatric epilepsy populations studied, and in some analyses showed lower seizure freedom rates. While LEV is widely used as first-line or early add-on therapy, these findings suggest that its comparative efficacy is generally similar to, and in some settings lower than, that of established ASMs. Findings addressing comparative seizure outcomes, however, do not provide a comprehensive evaluation of factors influencing treatment choices, which may also include reduced need for laboratory monitoring, lower potential for drug–drug interactions, availability of multiple formulations, ease of titration, and individual tolerability. Advantages LEV may offer in terms of drug-drug interactions should be weighed against its comparative effectiveness and its potential behavioral adverse effects.^41^ Although drug-drug interactions are relevant at all ages, they are considerably more problematic in the adult and elderly populations. Treatment decisions should therefore consider syndrome-specific evidence alongside patient-specific considerations.

Additional safety considerations such as longer-term tolerability, behavioral adverse effects, and reproductive safety remain important when selecting ASMs, particularly in adolescents who may require treatment into their reproductive years. Thus, while our analysis focuses on comparative seizure outcomes, ASM choice in routine care necessarily integrates these broader, patient-specific factors.

Our literature search identified 1,166 potentially relevant publications, of which only 25 (2.1%) were RCTs including pediatric patients. Beyond these RCTs, much of the available literature consists of small observational or uncontrolled studies, which may contribute to uncertainty regarding the comparative efficacy of LEV across pediatric epilepsy syndromes.^42^ Guidelines and essential medicine lists necessarily consider factors beyond efficacy, including accessibility, safety, and feasibility across healthcare settings.^43^ For example, while the National Institute for Health and Care Excellence NG217 guideline,^44^ acknowledges that LEV is not consistently more effective than older ASMs for seizure remission, it also highlights other factors such as tolerability, ease of administration, and economic considerations when positioning LEV alongside other treatment options. Although it is impossible to prove whether the high number of LEV prescriptions in clinical practice reflects the right balance between efficacy and the influence of these factors on therapeutic choices, our findings help contextualize these recommendations by providing comparative efficacy estimates derived from randomized trials.

In a broader perspective, the group of new ASMs to which LEV belongs has not been shown to be more effective than older drugs, but considered to offer practical advantages that may influence prescribing decisions in routine clinical care.^45^ However, the broad-spectrum role attributed to LEV in the pediatric population, which is not consistently supported by comparative randomized evidence and syndrome specific studies, could reflect an excessive influence of factors that are not evidence-based. After market authorization, comparative effectiveness data in specific pediatric epilepsy syndromes often remain limited. As a result, prescribing patterns may evolve based on a combination of personal experience, practical considerations, and extrapolation from adult or placebo/no-therapy–controlled studies. Our findings highlight the need for continued investment in pragmatic high-quality, syndrome-specific comparative trials to better inform treatment selection in children.

This study has several limitations. First, the overall quality of the included trials was variable, with only a minority judged to be at low risk of bias, and several showing concerns related to outcome measurement and selective reporting. Second, several included trials enrolled mixed-age populations extending into adulthood. Although this factor may limit the generalizability of some findings to strictly pediatric populations, our sensitivity analyses restricted to pediatric-only trials yielded consistent results. Third, follow-up duration differed across trials and was closely linked to the epilepsy subtype studied; although we stratified analyses by syndrome and excluded infantile spasms trials from the main analyses, heterogeneity in time horizons remains a limitation. Fourth, the comparator-based stratification resulted in placebo/no-therapy–controlled trials being predominantly adjunctive studies and active-comparator–controlled trials being predominantly monotherapy studies, which may limit direct comparisons across treatment contexts. Finally, our results suggested publication bias in placebo/no-therapy–controlled trials. Together, these factors may limit the precision and reliability of our pooled estimates and highlight the need for more pragmatic high-quality, syndrome-specific, head-to-head randomized trials in pediatric epilepsy. As with any evidence synthesis, real-world observational data were not included and may provide additional insights into treatment effectiveness and tolerability.

Clinicians should consider LEV as one of several therapeutic options in pediatric epilepsy, balancing its practical advantages against comparative efficacy data and potential behavioral adverse effects. Treatment decisions are best guided by epilepsy syndrome, patient-specific factors, and available evidence, rather than by default preference for any single medication.

## Glossary

ACTH: adrenocorticotropic hormone
ASM: antiseizure medication
CBZ: carbamazepine
CC: complete-case
IGE: idiopathic generalized epilepsy
ITT: intention-to-treat
LEV: levetiracetam
LTG: lamotrigine
PRISMA: Preferred Reporting Items for Systematic reviews and Meta-Analyses
RCT: randomized controlled trial
RD: risk difference
RoB-2: Risk of Bias 2
SeLECTS: self-limited epilepsy with centrotemporal spikes
SV2A: synaptic vesicle protein 2A
VPA: valproate
